# Melatonin Efficacy in Obese Leptin-Deficient Mice Heart

**DOI:** 10.3390/nu9121323

**Published:** 2017-12-05

**Authors:** Alessandra Stacchiotti, Gaia Favero, Lorena Giugno, Igor Golic, Aleksandra Korac, Rita Rezzani

**Affiliations:** 1 Anatomy and Physiopathology Division, Department of Clinical and Experimental Sciences, University of Brescia, Viale Europa 11, 25123 Brescia, Italy; alessandra.stacchiotti@unibs.it (A.S.); gaia.favero@unibs.it (G.F.); lorena.giugno@unibs.it (L.G.); 2 Interdipartimental University Center of Research “Adaptation and Regeneration of Tissues and Organs (ARTO)”, University of Brescia, 25123 Brescia, Italy; 3 Center for Electron Microscopy, Faculty of Biology, University of Belgrade, Studentski trg 16, 11000 Belgrade, Serbia; igor.golic@bio.bg.ac.rs (I.G.); aleksandra.korac@bio.bg.ac.rs (A.K.)

**Keywords:** leptin-deficient mice, melatonin, mitochondria, lipid peroxidation, pericardial fat

## Abstract

Cardiomyocytes are particularly sensitive to oxidative damage due to the link between mitochondria and sarcoplasmic reticulum necessary for calcium flux and contraction. Melatonin, important indoleamine secreted by the pineal gland during darkness, also has important cardioprotective properties. We designed the present study to define morphological and ultrastructural changes in cardiomyocytes and mainly in mitochondria of an animal model of obesity (*ob*/*ob* mice), when treated orally or not with melatonin at 100 mg/kg/day for 8 weeks (from 5 up to 13 week of life). We observed that *ob*/*ob* mice mitochondria in sub-sarcolemmal and inter-myofibrillar compartments are often devoid of cristae with an abnormally large size, which are called mega-mitochondria. Moreover, in *ob*/*ob* mice the hypertrophic cardiomyocytes expressed high level of 4hydroxy-2-nonenal (4HNE), a marker of lipid peroxidation but scarce degree of mitofusin2, indicative of mitochondrial sufferance. Melatonin oral supplementation in *ob*/*ob* mice restores mitochondrial cristae, enhances mitofusin2 expression and minimizes 4HNE and p62/SQSTM1, an index of aberrant autophagic flux. At pericardial fat level, adipose tissue depot strictly associated with myocardium infarction, melatonin reduces adipocyte hypertrophy and inversely regulates 4HNE and adiponectin expressions. In summary, melatonin might represent a safe dietary adjuvant to hamper cardiac mitochondria remodeling and the hypoxic status that occur in pre-diabetic obese mice at 13 weeks of life.

## 1. Introduction

Adult obesity is a worldwide pandemic condition that leads to the metabolic syndrome and a spectrum of irreversible cardiovascular, neurological, renal and digestive morbidities [1,2]. Nevertheless, cardiovascular diseases still represent the leading cause of death in obesity linked to hypertension, diastolic dysfunction and hypercoagulability [3,4]. Recent studies aimed to discover the pathogenesis of heart failure have underlined the crucial involvement of mitochondria dysfunctions that may precede the myocardial damage [5,6]. Indeed, the therapeutic role of drugs able to specifically target mitochondria dysfunctions is an intriguing and promising field also of cardiovascular research [7,8]. Although, to best address combined cellular events that concur in obesity, diabetes and related-cardiovascular dysfunctions, rodent models are still the best choice [9]. To adopt an experimental in vivo approach to mimic human obesity there are two main different translational models: genetically- or dietary-induced obesity. The major advantage of genetically induced obese mice, lacking either the leptin-gene (*ob*/*ob* mice) or the leptin gene receptor (*db*/*db* mice), is that they have been rigorously characterized and manifest cardiac dysfunctions and diabetes in a time-dependent manner [10,11]. In the present study, we focus on pre-diabetic leptin-deficient mice (the *ob*/*ob* mice model), obtained by a single point mutation of leptin gene. Leptin is an adipokine produced by the adipose tissue, strictly related to the level of adiposity and able to modulate food intake [12]. In healthy conditions, proper leptin signaling alerts the central nervous system, mainly the hypothalamic nuclei, to limit food intake and modulate energy expenditure [13]. Remarkably, exogenous leptin administration greatly ameliorates metabolic dysfunctions, oxidative damage, insulin resistance and over-weight in *ob*/*ob* mice [14].

The myocardium is an intricate and complex tissue where mitochondria constitute more than 45% of the total volume, providing energy and a spatial arrangement to calcium (Ca^2+^) delivery for a synchronous rhythm [15,16]. In adult cardiomyocytes, the mitochondria have been localized, by ultrastructural analysis, in three different compartments: perinuclear, inter-myofibrillar (IFM) and sub-sarcolemmal (SSM) [17]. Generally, SSM mitochondria, due to their peculiar position, are involved in overt production of reactive oxygen species and are more vulnerable to inhibition of the oxidative damage by excessive Ca^2+^ loading but also respond to cardioprotective drugs [18]. On the contrary, IFM mitochondria are more susceptible to apoptotic death in experimental diabetic condition [19]; whereas, perinuclear mitochondria are an independent population mainly involved in transcription and translation activities [17]. Nevertheless, mitochondria are hallmark of heart failure and many evidences indicate that they change shape and related metabolism in cellular death [20]. In the heart, during metabolically active conditions to restore homeostasis, mitochondria may shift from discrete round shape to an elongated network but abnormal remodeling and excessive shift between different sizes is a sign of heart damage [21]. Specific proteins modulate mitochondrial fusion or fission states like mitofusins and their genetic ablation in cardiomyocytes is lethal [22]. To remove abnormal or dysfunctional mitochondria, the heart uses a selective autophagic mechanism—mitophagy—essential for maintaining heart efficiency [23]. P62/SQSTM1 is a crucial player in the final step of autophagy involved in the definitive removal of auto-phagolysosomes deposits from the cellular environment, so it indicates proper or dysfunctional autophagic flux in the heart [24].

Previous studies reported in ventricular cardiomyocytes of *ob*/*ob* mice: hypertrophy, abnormal energy metabolism, aberrant intracellular Ca^2+^ handling and contractile systolic and diastolic dysfunctions [25,26]. In a recent study, Fruhbeck and coworkers [14] demonstrated that a low physiological dose of leptin administered in *ob*/*ob* mice reduced oxidative stress and inflammation through the elevation of adiponectin serum level. Adiponectin, an anti-inflammatory adipokine secreted almost exclusively by the adipocytes, is downregulated in cardiovascular inflammation and obesity [27]. This adipokine is also a vasodilator agent scarcely expressed in perivascular fat of obese patients and *ob*/*ob* mice [28].

Melatonin (*N*-acetyl-5-methoxytryptamine), the main product of the pineal gland, is a pleiotropic antioxidant and anti-inflammatory indoleamine with cardioprotective properties [29,30]. This lipophilic indoleamine easily crosses biological membranes and resides in mitochondria, where it prevents inner membrane alterations and inhibits permeability transition pore opening [31,32,33].

The aim of the present study was to assess the effect of oral supplementation of melatonin on cardiomyocytes, mainly on mitochondria and pericardial fat in pre-diabetic leptin-deficient *ob*/*ob* mice. Our results showed, at heart level, the complex mitochondrial alterations that occur during obesity and, remarkably, we suggested that melatonin might be a safe preventive dietary adjuvant able to impair cardiac hypoxic status in pre-diabetic leptin-deficient mice.

## 2. Materials and Methods

### 2.1. Animal Treatment

All procedures were performed according to the National Ministerial guidelines and were approved by the Italian Ministry of Health to comply with the commonly-accepted “3 Rs” indication. Male mice lean (B6.V^lean^/OlaHsd) and leptin-deficient (B6.V-Lep^ob^/OlaHsd) at 4 weeks of age were purchased from Harlan Laboratories Srl (San Pietro al Natisone, Udine, Italy). We treated 4 groups of 10 mice each as follows: (1) lean mice fed standard rodent diet and tap water (lean); (2) lean mice fed standard rodent diet and treated with melatonin in drinking water (lean + melatonin); (3) leptin-deficient mice fed standard rodent diet and tap water (*ob*/*ob*) and (4) leptin-deficient mice fed standard rodent diet and treated with melatonin in drinking water (*ob*/*ob* + melatonin). Melatonin was added to tap water from 5th to 13th weeks of life in drinking water at a final dose of 100 mg/kg/day. Details on melatonin preparation, rodent diet composition, water and food consumption and body weight gain have been previously reported [34,35]. At the end of the treatments, animals were sacrificed by cervical dislocation, heart and pericardial fat was removed and processed for histomorphometrical, immunohistochemical and ultrastructural evaluations, as previously described [36].

### 2.2. Histomorphometrical Evaluation

Serial heart paraffin sections (7 µm thick) of each sample were cut with a microtome stained with haematoxylin-eosin and then observed using an optical light microscope (Olympus, Hamburg, Germany) at a final magnification of 400×. At least 20 cardiomyocytes for five different slides were randomly examined for each experimental group to measure the short axis length obtained at level of nuclei using an image analysis program (Image Pro Plus, Milan, Italy). We decided to ignore to measure the total length of cardiomyocytes that can vary and it is hardly defined [37].

### 2.3. Alizarin S Red Staining

Briefly, after incubation in 1% sodium hydroxide in absolute ethanol for 30 min at 37 °C and rehydration with decreasing concentrations of ethanol, semi-thin sections (2 µm thick) of ventricular tissue Epon embedded were incubated in Alizarin Red S mixture for 5 min followed by acetone, acetone/xylene and xylene and finally the sections were mounted. Slides were observed under a Leica DMLB microscope (Leica Microsystems, Wetzlar, Germany) at final magnification of 400×. Alizarin Red S staining shows the presence and localization of Ca^2+^ in the heart by forming insoluble chelates with Ca^2+^ and gives an intense orange to red staining [38]. An observer without knowledge of the type of treatment undergone performed quantitative analysis of staining intensity.

### 2.4. Nuclear Cardiomyocyte Morphometry

Semi thin heart sections (1 µm thick) were stained with an aqueous mixture of Methylene blue and Azur II (1:1) on a hot plate for 1 min then washed in distilled water and were observed, at a final magnification of 1000× with an optical light microscope (Olympus, Hamburg, Germany) equipped with an image analysis program (Image Pro Plus, Milan, Italy). Major nuclear axis length, nuclear area and indentations—a peculiar site of Ca^2+^ flux according to Shimojima and coworkers [39]—were evaluated on 20 randomly chosen cardiomyocytes of five different slides for each experimental group. An observer without knowledge of the type of treatment undergone performed quantitative analysis of staining intensity.

### 2.5. Mitochondria Evaluation

Ultra-thin sections (80 nm thick) of ventricular tissue were obtained using a Leica UC6 ultramicrotome (Leica Microsystems, Wetzlar, Germany), mounted on copper grids and contrasted in uranyl acetate and lead citrate using Leica EM STAIN (Leica Microsystems, Wetzlar, Germany). Grids were examined on a Philips CM12 transmission electron microscope (Philips/FEI, Eindhoven, The Netherlands) equipped with the digital camera SIS MegaView III (Olympus Soft Imaging Solutions, Munster, Germany). The obtained electron photomicrographs were used for both ultrastructural and morphometric analysis of mitochondria. Mitochondrial mean diameter was calculated as the average diameter (obtained by combining diameter 1 and diameter 2 measures) for each mitochondrion, using the Image J software (Image Processing and Analyses in Java, NIH, Bethesda, MD, USA) and then mitochondria were divided in appropriate classes in dependence of their diameter. 200 SSM and 200 IFM mitochondria were randomly observed for each experimental group in almost 3 mice/group, at a final magnification of 13,000×. We excluded from the quantitative analysis perinuclear mitochondria because they are organized as clusters that make difficult to correctly analyze a single unit [40].

### 2.6. Immunohistochemical Analysis

Serial heart paraffin sections (7 µm thick) were dewaxed in xylene, rehydrated and subjected to antigen retrieval in 0.01M citrate buffer (pH 6.0) in a microwave oven for subsequent cycles of 5 min at 700 Watt and a cycle of 3 min at 500 Watt, then treated in 3% hydrogen peroxide to block endogenous peroxidase staining. Then slides were immersed in normal goat or horse serum according to the species producing the secondary antibody (1:5 dilution) for 1 hour at room temperature in the dark and subsequently, without any washing, with primary antibodies. In the present study, we evaluated the following primary antibodies: monoclonal mouse antibodies against mitofusin2 (Mfn2) clone 6A8 (Abnova, Taipei, Taiwan) diluted 1:200; polyclonal rabbit antibodies against adiponectin (Abcam, Cambridge, UK) diluted 1:500; rabbit polyclonal antibodies against p62/SQSTM1 (MBL Ltd, Jasola, New Delhi, India) diluted 1:50 and rabbit polyclonal antibodies against 4 hydroxy-2-nonenal (4HNE) (Abcam, Cambridge, UK) diluted 1:400. After washing in tris buffer saline (TBS), the sections were incubated with the avidin-biotin-peroxidase complex (ABC-peroxidase kit, Vector Labs, Burlingame, CA, USA), according to manufacturer instruction. Finally, the peroxidase reaction was developed using 3′-3′-diaminobenzidine tetrahydrochloride (Sigma Aldrich, St. Louis, MO, USA) as substrate and hydrogen peroxide as catalizator for 8 min in the dark. Negative controls were performed for each set of experiments by omitting primary antibodies and substituting them with TBS. All sections were counterstained with haematoxylin, dehydrated and mounted for the analysis with an optical light microscope (Olympus, Hamburg, Germany). Two observers without knowledge of the type of treatment undergone assessed immunopositivity—expressed as arbitrary units (AU)—of each primary antibody. Almost 25 random fields, with the same area, for each primary antibody, were estimated using an image analysis program (Image Pro Plus, Milan, Italy).

### 2.7. Statistical Analysis

All results were indicated as mean ± SEM. Analysis of the statistical significance was performed using a one-way analysis of variance-ANOVA corrected by Bonferroni test to compare the variability of a group with all other experimental groups. *p* ≤ 0.05 was set as significant value. All experiments were carried out in triplicate, data collected and analyzed by Origin Pro 9.1 software (OriginLab Coorporation, Northampton, MA, USA).

## 3. Results

Experimental treatment with exogenous melatonin dissolved in drinking water for 8 weeks were well tolerated and all animals survived up to 13 weeks of age. This finding largely confirms the safe nature of melatonin supplementation and the absence of overt side effects [41]. As previously reported by our research group, in *ob*/*ob* leptin-deficient mice the metabolic data and blood glucose were elevated but significantly reduced by melatonin treatment, even if less extent than in lean groups [34,35]. Lean mice treated or not with melatonin displayed similar “normal” metabolic and glycemic data, so they are defined generically as “lean” mice in the following histomorphometrical, immunohistochemical and ultrastructural analyses.

**Figure 1:**
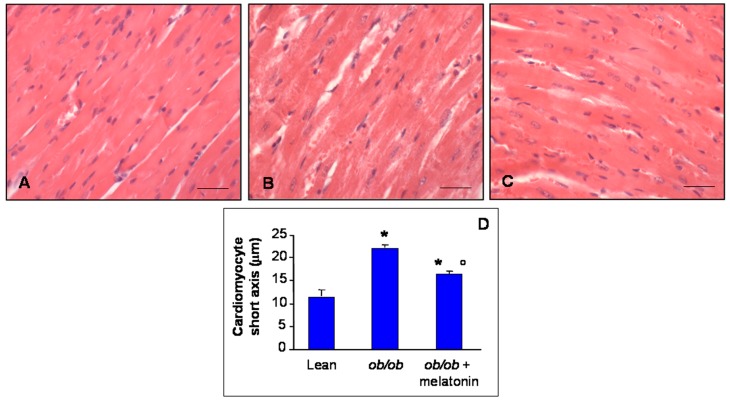
Histomorphometrical evaluation. Photomicrographs showing ventricular cardiomyocytes of lean mice treated and not treated with melatonin (**A**), *ob*/*ob* mice (**B**) and *ob*/*ob* mice treated with melatonin (**C**). Lean mice treated or not with melatonin displayed similar “normal” morphological data, so they are defined generically as “lean” mice. Note in *ob*/*ob* mice supplemented with melatonin there is a partial restoration of proper cytoarchitecture in longitudinal heart sections. Haematoxylin-eosin staining. Scale Bar = 20 µm. The graph (**D**) summarized the analyses of ventricular cardiomyocytes short axis. * *p* ≤ 0.05 significant vs. lean mice; ° *p* ≤ 0.05 significant vs. *ob*/*ob* mice.

The histomorphometrical analysis of ventricular cardiomyocytes revealed a longitudinal well-organized array of cells—generally mononucleated—with normal borders in heart lean mice (Figure 1A). In contrast, *ob*/*ob* mice showed misaligned cardiomyocytes with an irregular contour and often doubled nuclei (Figure 1B). However, after melatonin supplementation, *ob*/*ob* mice heart cytoarchitecture ameliorated significantly, even if binucleated cardiomyocytes persisted (Figure 1C). Estimation of short cardiomyocyte axis indicated ventricular hypertrophy in *ob*/*ob* mice (22.2 ± 0.3 µm) compared to lean mice (11.6 ± 0.4 µm) and limited hypertrophy with an intermediate size for *ob*/*ob* mice treated with melatonin (16.1 ± 0.4 µm) (Figure 1D).

**Figure 2:**
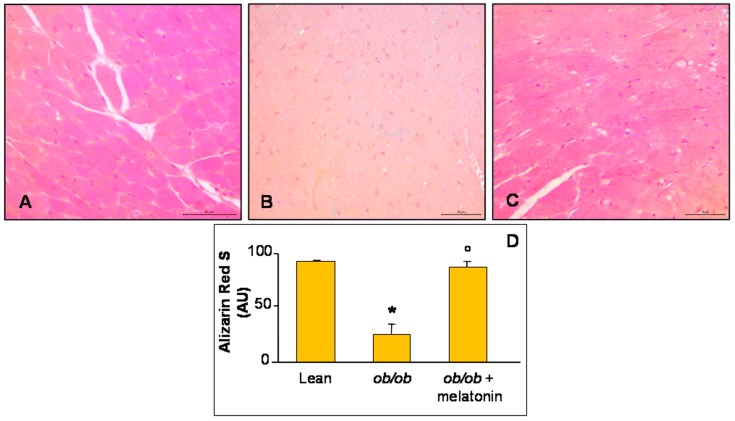
Alizarin Red S staining evaluation. Photomicrographs showing Ca^2+^ accumulation in ventricular cardiomyocytes of lean mice treated and not treated with melatonin (**A**), *ob*/*ob* mice (**B**) and *ob*/*ob* mice treated with melatonin (**C**). Lean mice treated or not with melatonin displayed similar “normal” cardiomyocytes Ca^2+^ accumulation data, so they are defined generically as “lean” mice. Note a bright orange to red Ca^2+^ salts presence in lean mice, whereas a decreased accumulation in *ob*/*ob* mice and a significant recovery after melatonin treatment. Alizarin Red S staining. Scale Bar = 50 µm. The graph (**D**) summarize the quantitative analysis of Alizarin Red staining. * *p* ≤ 0.05 significant vs. lean mice; ° *p* ≤ 0.05 significant vs. *ob*/*ob* mice.

Furthermore, histopathological assessment of Ca^2+^ salts deposition in all the experimental groups was carried out and, respect to lean group that showed a moderate/strong ions deposition (Figure 2A), *ob*/*ob* mice heart has a strong reduction in Ca^2+^ accumulation (Figure 2B). A significant recovery after melatonin intake was observed, almost similar to lean mice (Figure 2C). These observations are confirmed also by quantitative analyses summarized in Figure 2D.

**Figure 3:**
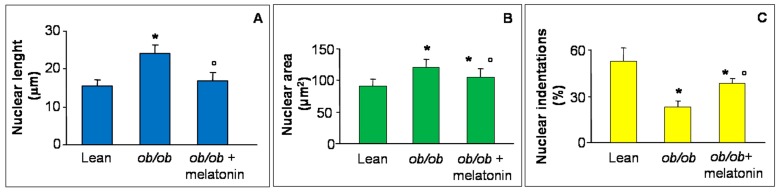
Nuclear features of cardiomyocytes. The graphs summarize the cardiomyocyte nuclear features analyzed: major axis length (**A**), area (**B**) and indentation (**C**) of all the experimental groups. Quantification of major nuclear axis and nuclear area indicated a strong increase of both parameters in *ob*/*ob* mice, that appeared both attenuated after melatonin treatment. Lean mice treated or not with melatonin displayed similar “normal” nuclear features of cardiomyocytes, so they are defined generically as “lean” mice. Note that nuclear indentations decreased in *ob*/*ob* mice but enhanced after melatonin intake. * *p* ≤ 0.05 significant vs. lean mice; ° *p* ≤ 0.05 significant vs. *ob*/*ob* mice.

Disrupted Ca^2+^ flux has been recently reported in hypertrophic rat cardiomyocytes morphologically linked to abnormal nuclear size and loss of indentations, specific sites for Ca^2+^ nuclear transition and a crucial early event in heart failure [39,40,41]. For this reason, in the present study, we analyzed major nuclear axis length (Figure 3A), nuclear area (Figure 3B) and nuclear indentations along nuclear contour (Figure 3C) in all the experimental groups. In detail, both nuclear length and area increased in *ob*/*ob* mice cardiomyocytes in comparison to lean mice and decreased after melatonin supplementation. In contrast, indentations percentage significantly decreased in *ob*/*ob* mice but enhanced in *ob*/*ob* mice treated with melatonin, so corroborating the beneficial melatonin effect on Ca^2+^ flux at heart level. For the relative photomicrographs of representative fields, see Figure S1.

**Figure 4:**
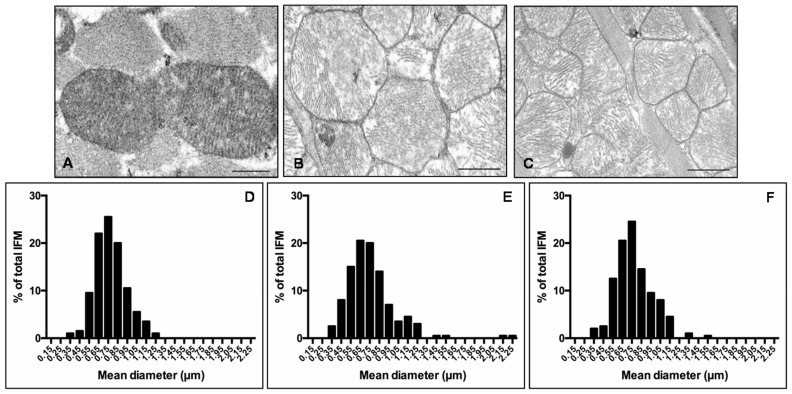
Inter-myofibrillar heart mitochondria features. Photomicrographs showing heart mitochondria of lean mice (**A**), *ob*/*ob* mice (**B**) and *ob*/*ob* mice treated with melatonin (**C**). Representative ultrastructural photomicrographs of IFM mitochondria closely packed inside myofibrils of sarcomeres. Scale Bar = 500 nm. The graphs summarize the IFM mitochondria diameter and distribution along size-classes of lean mice (**D**), *ob*/*ob* mice (**E**) and *ob*/*ob* mice treated with melatonin (**F**). Lean mice treated or not with melatonin displayed similar “normal” nuclear features of cardiomyocytes, so they are defined generically as “lean” mice.

Given that heart is the most mitochondria-rich organ of the body and bases on an oxidative metabolism, many studies reported that mitochondria morphology and function are impaired in cardiovascular diseases and obesity [22,42,43]. Consequently, we decided to analyses cardiac mitochondria sub-populations, mainly IFM and SSM mitochondria, by transmission electron microscopy. IFM mitochondria of lean mice presented well-organized cristae and dense matrix (Figure 4A) compared with IFM mitochondria of *ob*/*ob* mice that were heterogeneous in shape with the tendency to form “mega-mitochondria,” often devoid of cristae (Figure 4B). Interestingly, melatonin treated *ob*/*ob* mice presented regular mitochondria and ameliorated inner membrane organization (Figure 4C). Quantification of IFM mitochondria diameter and distribution along size-classes indicated a homogeneous distribution of mitochondria diameter in lean mice (Figure 4D), whereas, a larger mitochondrial size and the onset of extreme classes like very small (<450 nm) or very large (>1.05 µm) were observed in *ob*/*ob* mice (Figure 4E). Remarkably, in *ob*/*ob* mice treated with melatonin the values shifted into intermediate values (750–850 nm) similar to lean mice (Figure 4F). Similar observations were obtained evaluating SSM heart mitochondria (see Figure S2).

**Figure 5:**
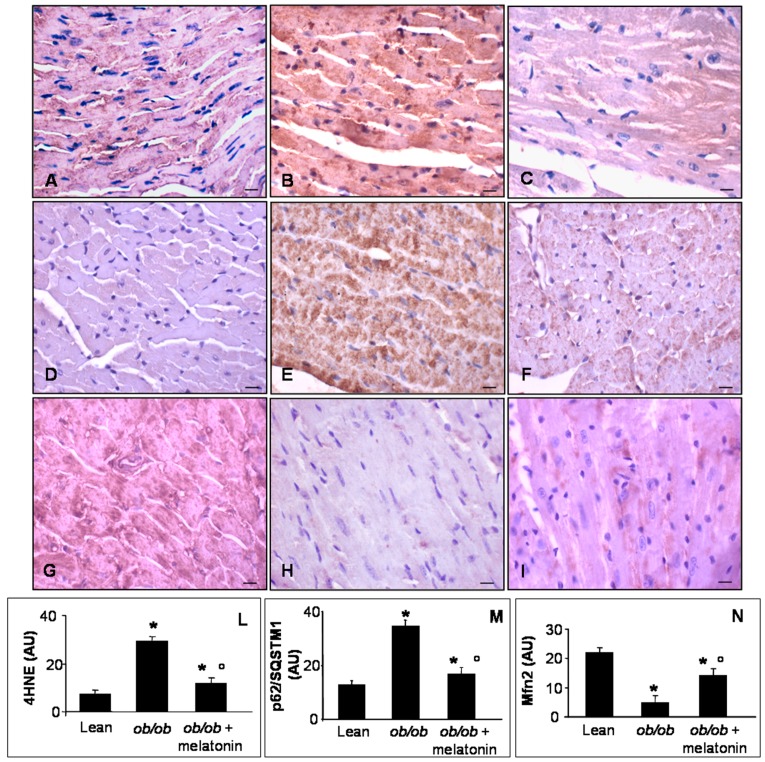
Heart lipid peroxidation, autophagy and mitochondrial health markers evaluation. Photomicrographs showing heart 4HNE (**A**–**C**), p62/SQSTM1 (**D**–**F**) and Mfn2 (**G**–**I**) immunostainings of lean mice treated and not treated with melatonin (**A**,**D**,**G**), *ob*/*ob* mice (**B**,**E**,**H**) and *ob*/*ob* mice treated with melatonin (**C**,**F**,**I**). Lean mice treated or not with melatonin displayed similar “normal” heart lipid peroxidation, autophagy and mitochondrial health markers, so they are defined generically as “lean” mice. Note that melatonin treatment attenuated mitochondrial oxidative damage linked to obesity. Scale bars = 20 µm. The graphs summarize the 4HNE (**L**), p62/SQSTM1 (**M**) and Mfn2 (**N**) immunohistomorphometrical analyses. Relative immunopositivities indicate a significant attenuation of the anomalous expression of the above markers in *ob*/*ob* mice treated with melatonin. * *p* ≤ 0.05 significant vs. lean mice; ° *p* ≤ 0.05 significant vs. *ob*/*ob* mice.

Lipid peroxidation, indicated by 4 hydroxy-2-nonenal (4HNE) adducts, has been well characterized in mitochondria of cardiovascular metabolic diseases and in pre-diabetic status induced by high fatty diet or low dose of streptozotocin in mice [44]. Mfn2 is a dynamin-like protein involved in the organization of mitochondrial network in cardiomyocytes and necessary for Ca^2+^ transfer between sarcoplasmic reticulum and mitochondria [44,45,46]. P62/SQSTM1, called also sequestosome-1-SQSTM1, is a regulator of cardiac autophagy, a crucial mechanism to adapt cardiomyocytes to toxic and metabolic stress [23,47]. For all these reasons, we decided to analyze 4HNE, Mfn2 and p62/SQSTM1 expressions at heart level. In detail, lean mice showed scarce 4HNE and p62/SQSTM1 immunostainings (Figure 5A,D), whereas *ob*/*ob* mice presented abnormal intense 4HNE and p62/SQSTM1, expressions (Figure 5B,E). Melatonin supplementation in *ob*/*ob* mice was able to attenuate both these dangerous signals in cardiomyocytes (Figure 5C,F). Remarkably, Mfn2 was highly expressed in heart of lean mice (Figure 5G) and dramatically decreased in *ob*/*ob* mice (Figure 5H) but was partially restored in *ob*/*ob* mice treated with melatonin (Figure 5I). These observations were confirmed also by immunohistomorphological analyses of heart 4HNE (Figure 5L), p62/SQSTMI (Figure 5M) and Mfn2 (Figure 5N). Taken together these results indicated a significant antioxidant effect of melatonin oral supplementation in *ob*/*ob* mice, greatly addressed to restore mitochondrial health in cardiomyocytes.

**Figure 6:**
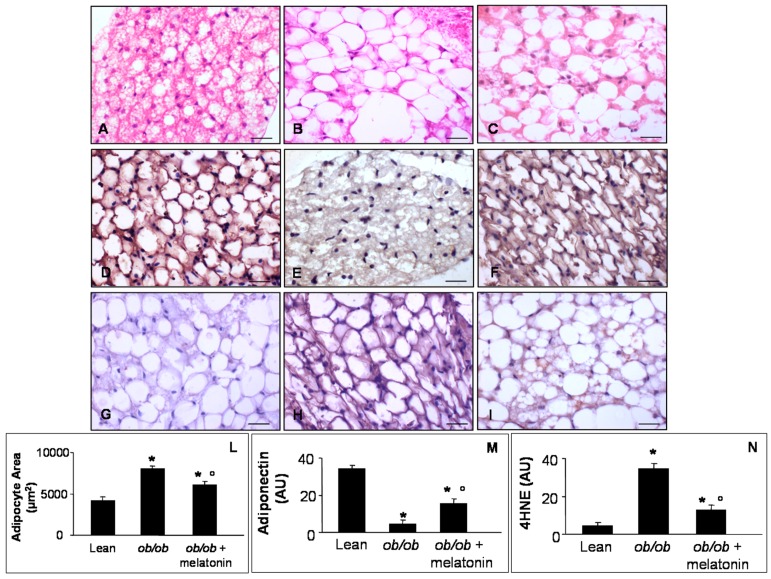
Pericardial adipose tissue evaluations. Photomicrographs showing pericardial adipocytes morphological feature through haematoxylin-eosin staining (**A**–**C**), anti-obesogenic adiponectin (**D**–**F**) and 4HNE (**G**–**I**) immunostainings of lean mice treated and not treated with melatonin (**A**,**D**,**G**), *ob*/*ob* mice (**B**,**E**,**H**) and *ob*/*ob* mice treated with melatonin (**C**,**F**,**I**). The graphs summarize adipocyte area (**L**), adiponectin (**M**) and 4HNE (**N**) immunopositivities. Lean mice treated or not with melatonin displayed similar “normal” pericardial adipose tissue features, so they are defined generically as “lean” mice. Note that *ob*/*ob* mice treated with melatonin showed a reduced white adipocytes area and inverse relationship between anti-diabetogenic adiponectin and 4HNE. Scale Bar = 20 µm. * *p* ≤ 0.05 significant vs. lean mice; ° *p* ≤ 0.05 significant vs. *ob*/*ob* mice.

A controversial role has been attributed to the pericardial fat in cardiac damage linked to obesity, due to nomenclature, hard visualization [48] and interspecies differences, i.e., the epicardial fat is absent in rodents but present in patients [49]. However, we evaluated pericardial adipocytes, measuring their area (Figure 6A–C) and the expression of anti-obesogenic and oxidative stress markers: adiponectin (Figure 6D–F) and 4HNE (Figure 6G–I). In detail, lean mice showed regular small adipocytes (Figure 6A), whereas *ob*/*ob* mice showed a higher adipocyte area (Figure 6B). Interestingly, melatonin treatment of *ob*/*ob* mice reduced significantly white adipocyte area (Figure 6C). Moreover, lean mice showed a moderate/strong adiponectin expression (Figure 6D) and a weak 4HNE immunopositivity (Figure 6G) compared with respectively weak and moderate/strong expressions in *ob*/*ob* mice pericardial adipose tissue (Figure 6E,H**)**. Melatonin supplementation adversely upregulated adiponectin (Figure 6F) but minimized 4HNE expressions in *ob*/*ob* mice (Figure 6I). Quantitative analysis confirmed these results (Figure 6M,N), corroborating anti-inflammatory and antioxidant effects of melatonin also at pericardial adipose tissue level.

## 4. Discussion

In the present study, it was shown that melatonin oral supplementation, at the dose of 100 mg/kg/day for 8 weeks, attenuated cardiac hypertrophy and adaptive remodeling in leptin-deficient (*ob*/*ob*) mice at 13 weeks of age, before overt insulin resistance. Anyway, in *ob*/*ob* mice supplemented with melatonin the cardiac dysfunctions did not completely disappeared. We are confident that major time of melatonin supply or a highest dose might be more effective to reverse cardiac changes. However, in the present study we are particularly interested to evaluate early cardiac melatonin effects in obese mice, so we ended treatments in mice of 13 weeks of age to avoid the occurrence of severe diabetic complications. Then focusing on cardiac mitochondria, we demonstrated that melatonin restored proper cristae feature, sustained Mfn2 expression and limited mega-mitochondria in different subcellular regions. Inner mitochondrial cristae are emerging, not only as centers for providing energy but also dynamic structures that change orientation/shape according to metabolic requirements in a mitochondrial network [50]. Intriguingly, in our experiments, melatonin induced the same beneficial effect in IFM and SSM mitochondria. This important finding might sound unusual, considering previous evidences on different roles of cardiac mitochondria associated to their different location and the influence on pathological status [17]. In particular, by scanning electron microscopy IFM mitochondria possess lamelliform cristae, more useful to produce ATP, in comparison to tubular cristae in SSM mitochondria [51]. However, SSM mitochondria contain elevated Ca^2+^ and sodium ions, generated by electric stimulation and better react to hypoxia [52]. A recent proteomic analysis in the heart of type 1 and type 2 diabetes demonstrated that both IFM and SSM mitochondrial populations loose inner membrane integrity [53]. The common beneficial effect of melatonin in both SSM and IFM mitochondria might effectively seem a paradox. Nevertheless, melatonin could act not only as a free radical scavenger but also as regulator of membrane fluidity and inter-mitochondrial contacts, leading to abrogation of mega-mitochondria. In previous experimental studies, melatonin preserved mitochondria by preventing the decrease in Ca^2+^ threshold and inner membrane dysfunctions and ATP production in cardiovascular diseases and in obese rats [31,32]. Our histomorphometrical data on Ca^2+^ deposition by Alizarin Red staining, besides the quantification of nuclear indentations in cardiomyocytes, indicated the ability of melatonin to recover Ca^2+^ levels and its influence on mitochondrial uncoupling, i.e., increased fatty acid oxidation but reduced efficiency in *ob*/*ob* mice [54]. Remarkably, mega-mitochondria described in *ob*/*ob* mice heart probably represented an adaptive reaction to unfavorable thermogenic oxidant environment [38,55] but they disappeared after melatonin treatment. Yang and coworkers [56] have reported dysfunctional autophagy in the liver of obese *ob*/*ob* mice and we recently demonstrated that exogenous melatonin reduced endoplasmic reticular stress and p62/SQSTM1 in the same animal model [36]. So, similar melatonin behavior in the obese heart confirmed the restoration of cardiomyocyte homeostasis, further corroborated by reduced ventricular hypertrophy [23].

4HNE has been previously indicated as a crucial signaling molecule in the regulation of mitochondrial uncoupling [57]. Moreover, lipid peroxidation may occur when cardiolipin, the unique inner mitochondrial lipid membrane, was altered during an oxidant event and induced mitochondrial dysfunctions [32]. Therefore, we reported a strong 4HNE reduction in *ob*/*ob* mice supplemented with melatonin in comparison to aberrant signal in the *ob*/*ob* strain. These findings allowed us to think that melatonin acted also as a consolidate cleaner of oxidized lipids and stabilizer of the inner mitochondrial membrane in *ob*/*ob* mice cardiomyocytes. Another interesting result is Mfn2 recovery in cardiomyocytes by daily melatonin intake for 8 weeks. Mfn2 was firstly discovered as a mutated fusion protein in the outer mitochondrial membrane in Charcot-Marie-Tooth type 2 neurodegeneration [58], even if it may function also as a mediator of autophagy [59]. Indeed, mice with Mfn2-specific heart ablation displayed high vulnerability to ischemia and mitochondrial damage [60]. Our research group has observed previously that melatonin supplementation significantly improved the obesity induced renal alterations, restoring mitochondrial shape and Mfn2 expression [61].

Finally, in this study we described the beneficial effects of melatonin supplementation on also pericardial fat. This finding was particularly interesting because low adiponectin correlated with impaired mitochondria and altered myocardial recovery after infarction in *ob*/*ob* mice, in type 2 diabetic patients and in streptozotocin-treated rats [62,63]. In contrast, in visceral and perivascular fat around small resistance arteries in *ob*/*ob* mice, melatonin supply ameliorated glycaemia, hampered abnormal fat depots, rescued anti-contractile activity [28,34]. We observed also that melatonin supplementation reduced large adipocytes and 4HNE expression in pericardial fat, so validating its efficacy on metabolic abnormalities [64].

## 5. Conclusions

**Figure 7:**
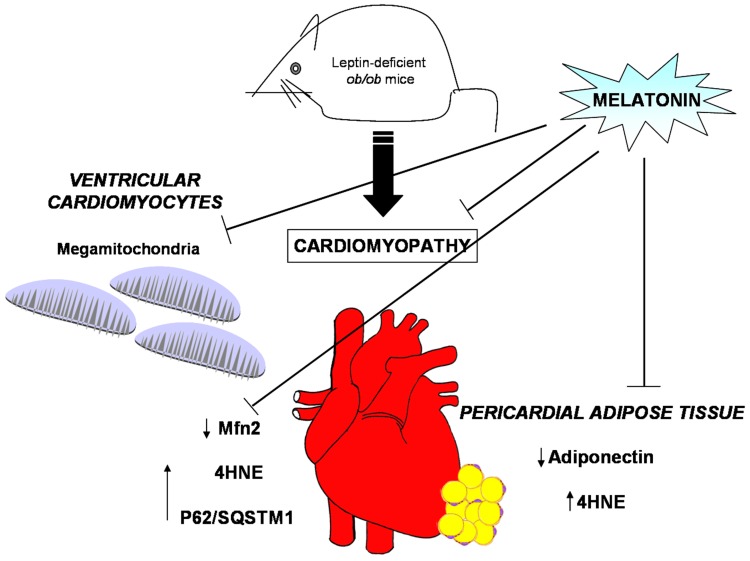
Obesity alterations and melatonin protective effects at both heart and pericardial adipose tissue level. Leptin deficient *ob*/*ob* mice presented, in ventricular cardiomyocytes, mitochondria with abnormal large size, called mega-mitochondria, together with high level of both 4hydroxy-2-nonenal (4HNE), a marker of lipid peroxidation and of p62/SQSTM1, an index of aberrant autophagic flux but scarce degree of mitofusin2 (Mfn2), indicative of mitochondrial sufferance. Furthermore, the *ob*/*ob* mice showed, in pericardial adipose tissue, low level of adiponectin expression and higher level of 4HNE, respect lean mice. Interestingly, melatonin oral supplementation in the heart restored mitochondria shape and ultrastructure, enhanced Mfn2 expression and minimized 4HNE and p62/SQSTM1. In addition, at pericardial fat level, melatonin reduced adipocyte hypertrophy, increased adiponectin and reduced 4HNE expressions.

Melatonin is an intriguing multitasking indoleamine, present also in edible food like vegetable and fish [65], that ameliorates mitochondrial and metabolic changes in the heart of adult genetically induced obese animal model (Figure 7). This translational study suggests that dietary melatonin supplementation might limit obesogenic changes in the heart and preserve mitochondrial size and membranes, so their proper energetic function.

## Supplementary Materials

The following are available online at www.mdpi.com/2072-6643/9/12/1323/s1, Figure S1: Nuclear cardiomyocytes morphology and ultrastructure, Figure S2: Heart sub-sarcolemmal mitochondrial features.
