# Supplementary material for: Melatonin Efficacy in Obese Leptin-Deficient Mice Heart
Source: Nutrients. 2017 Dec 5;9(12):1323. doi: 10.3390/nu9121323 (PMC5748773; doi:10.3390/nu9121323)
Supplement: Supplementary file 1 [file nutrients-09-01323-s001.pdf]

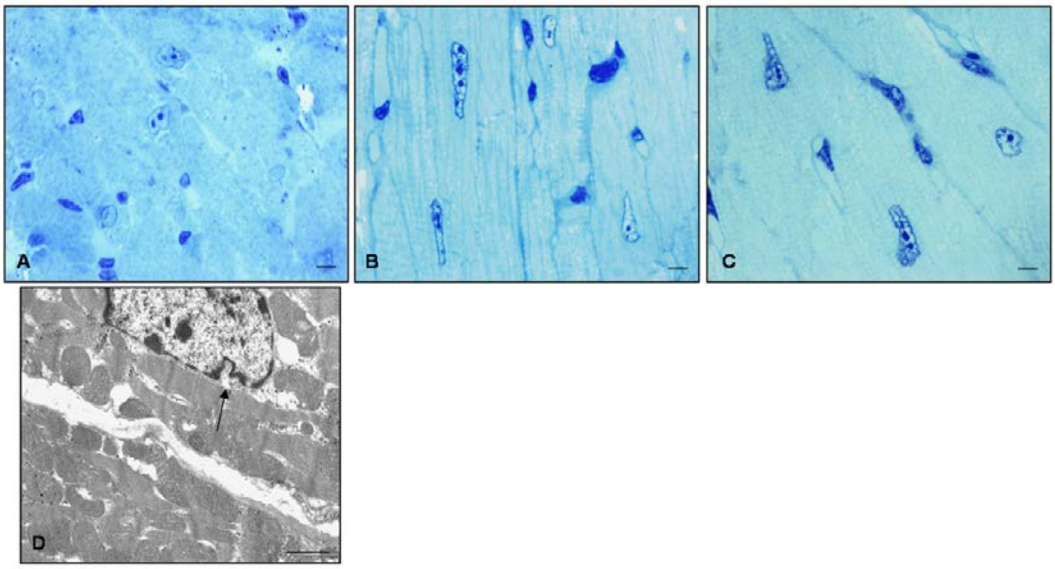

Figure S1. Nuclear cardiomyocytes morphology and ultrastructure. Photomicrographs showing **v**entricular cardiomyocytes in lean mice (**A**), *ob*/*ob* mice (**B**) and *ob*/*ob* mice treated with melatonin (**C**) presented different nuclear size and contour. Semithin sections stained by methylene blue-azur II. Ultrastructural evidence of nuclear indentations (arrow) in lean mice (**D**). Scale bars = A–C: 20 μm; D: 1 μm.

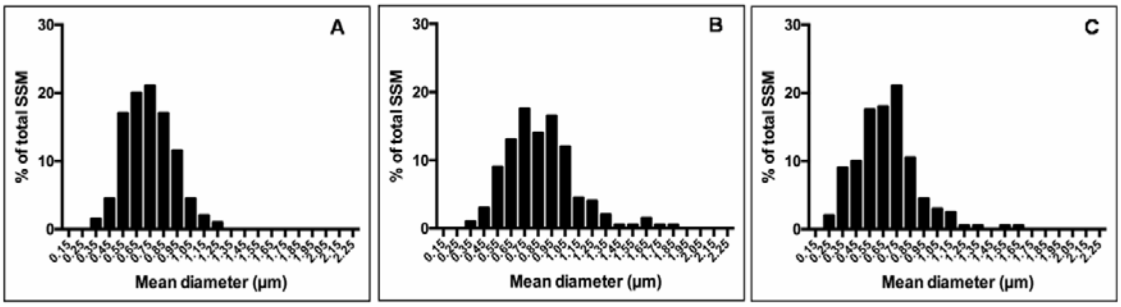

Figure S2. Heart sub-sarcolemmal mitochondrial features. The graphs summarize the SSM mitochondria diameter and distribution along size-classes of lean mice (**A**), *ob*/*ob* mice (**B**) and *ob*/*ob* mice treated with melatonin (**C**).
